# Elevated fecal calprotectin is linked to psychosocial complexity in pediatric functional abdominal pain disorders

**DOI:** 10.1186/s13104-021-05776-5

**Published:** 2021-09-15

**Authors:** Erin L. Moorman, Michael Farrell, Neha Santucci, Lee Denson, Christine Le, Natoshia R. Cunningham

**Affiliations:** 1 Department of Clinical and Health Psychology, University of Florida, P.O. Box 100165, Gainesville, FL 32610-0165 USA; 2 Division of Gastroenterology, Hepatology and Nutrition, Cincinnati Children’s Hospital Medical Center, Cincinnati, OH USA; 3 College of Medicine, University of Cincinnati, Cincinnati, OH USA; 4 Division of Behavioral Medicine and Clinical Psychology, Cincinnati Children’s Hospital Medical Center, Cincinnati, OH USA; 5 Department of Family Medicine, Michigan State University, Grand Rapids, MI USA

**Keywords:** Fecal calprotectin, Functional disability, Anxiety

## Abstract

**Objective:**

Children with functional abdominal pain disorders (FAPD) and clinical elevations in three risk areas (anxiety, functional disability, and pain) have been found to be at increased risk for persistent disability. We evaluated if the presence of these three risk factors corresponded with greater gastrointestinal inflammation (measured via fecal calprotectin; FC) compared to those with no risk factors. FC concentration differences between children with three risk factors and those with one and two risk factors were explored.

**Results:**

Fifty-six children with FAPD (*M*_age_ = 12.23) completed measures of anxiety (Screen for Child Anxiety Related Disorders), disability (Functional Disability Inventory), and pain intensity (Numeric Rating Scale). Participants were stratified into risk groups (range: 0–3). Fisher’s exact tests were conducted to determine if children with three versus fewer risk factors were more likely to have elevated FC (≥ 50 µg/g) versus normal levels. Children with three risk factors (*M*_FC_ = 86.04) were more likely to have elevated FC compared to children with zero (*M*_FC_ = 25.78), one (*M*_FC_ = 38.59), and two risk factors (*M*_FC_ = 45.06; *p*’s < 0.05). Those with three risk factors had borderline elevated FC concentrations whereas those with fewer had normal FC concentrations. Findings suggest the importance of a biopsychosocial approach to help elucidate a FAPD phenotype.

Functional abdominal pain disorders (FAPD) are gastrointestinal (GI) conditions characterized by frequent abdominal pain episodes not attributed to an organic cause [1]. FAPD occurs in approximately 11–15% of all children and adolescents worldwide [2], and 25–45% experience persistent (i.e., > 5 years) pain and functional impairment [3]. A biopsychosocial approach may be helpful for understanding and assessing pediatric FAPD [4–6], as the presence of certain psychosocial risk factors (i.e., anxiety) are associated with increased functional impairment in children with FAPD [7–9]. Further, those who present with clinical elevations in three domains (i.e., anxiety, pain, and pain-related disability) are at increased risk for persistent disability [7, 8]. However, it is unknown how biological factors, such as GI inflammation, may relate to this complex clinical presentation of FAPD.

Although FAPD are typically thought to be non-inflammatory [10–12], low-grade GI inflammation may be present, and may relate to greater functional disability [13]. The goal of the current study is to establish the relationship between psychological and biological factors associated with complex FAPD. We predicted that the most clinically complex FAPD group (i.e., those characterized by clinically elevated anxiety, disability, and pain levels) [7, 8] would have clinically elevated fecal calprotectin (FC) levels, a GI inflammatory biomarker, compared to those in the lowest risk group (i.e., those with no elevations in anxiety, disability, and pain). We also explored differences in FC concentrations based on numbers of risk factors (ranging from 0 to 3).

## Main text

### Methods

Clinical data were gathered from 56 children between the ages of 9–14 with FAPD from a large Midwestern children’s hospital using de-identified data as a part of an Institutional Review Board approved process. These youth were also involved in prior studies examining (1) clinical characteristics of youth with FAPD [7–9], (2) neuroimaging of pain, or (3) behavioral interventions for FAPD [14, 15]. The participant’s gastroenterologist confirmed that the patient met criteria for FAPD based on ROME IV guidelines and also performed an FC assay. Patients were ineligible if they had a significant medical condition(s) with an identifiable organic cause or a documented developmental delay. Participants did not have an active infection at the time of study enrollment.

### Measures

#### Demographic and background information

Demographic (e.g., age, sex) and background information (e.g., abdominal pain location) was collected from the parent and from the patient’s electronic medical record (EMR).

#### Fecal calprotectin (FC)

Fecal calprotectin is a biomarker of neutrophilic intestinal inflammation and is commonly assayed to evaluate a patient’s risk for inflammatory bowel disease (IBD) [16]. FC concentrations are obtained via a fecal assay and, due to a high negative predictive value to exclude IBD, have been used to differentiate IBD from functional GI conditions [17]. FC has greater sensitivity (70%) and specificity (93%) in children than adults [18]. To obtain FC concentrations, participants provided a stool sample as part of their clinical care. The Inova Quanta Lite^®^ Calprotectin ELISA assay was used to test FC levels. These values were recorded in the participants’ EMR and obtained via chart review. Manufacturer-recommended clinical cut-offs for interpreting FC concentrations include normal (< 50 µg/g), borderline (50–120 µg/g), and abnormal (> 120 µg/g).

#### Clinical risk

Participants were stratified into risk groups (range: 0–3) based on their scores on the measures detailed below. These measures were administered as part of their clinical care to determine risk status [7, 15]. Clinical elevations in all three domains listed below were defined as the most clinically complex FAPD group. This approach been shown to identify patients at greatest risk for pain-related impairment over time [7].

##### Screen for Child Anxiety Related Disorders—Child Report (SCARED) [19, 20]

A 41-item measure (total score range: 0–82) that assesses anxiety symptoms. It has been validated in pediatric chronic pain samples [21, 22] and is used to screen for anxiety in children with FAPD [8, 15]. A cut-off of ≥ 25 is indicative of clinically significant anxiety [19, 20] and has been shown to correspond to increased pain-related impairment and pain levels in youth with chronic pain including FAPD [7, 8, 14, 15]. Therefore, this cut-off was used to determine presence of risk in the current study.

##### Functional Disability Inventory—Child Version (FDI) [23]

A validated measure of daily functional impairment due to pain symptoms. The FDI consists of 15 items (total score range: 0–60) and has established cut-offs [24]. Participants with at least moderate disability (FDI ≥ 13) were considered clinically elevated.

##### Numeric Rating Scale (NRS) Pain Intensity [25]

Participants rated their average pain intensity over the past 2 weeks using a scale ranging from 0 to 10, with higher scores indicating higher pain levels. Participants reporting at least moderate pain (NRS ≥ 4) were considered clinically elevated.

#### Additional clinical data

Erythrocyte sedimentation rates (ESR), c-reactive protein (CRP), albumin, immunoglobulin A (IgA), and anti-tissue transglutaminase antibodies (tTG-IgA) are laboratory markers commonly obtained via blood test for differential diagnostic purposes in GI clinics [26, 27]. ESR and CRP are sometimes used by providers to help establish a diagnosis of IBD. ESR, the rate at which erythrocytes settle in a blood sample, indicates inflammation in the body and has been shown to correlate with clinical activity in individuals with IBD [28]. CRP is a protein produced by the liver that is released into the bloodstream often after inflammation onset [29]. It has also been associated with clinical activity in pediatric IBD, particularly with Crohn’s disease [29]. The tTG-IgA test is positive in about 98% of patients with celiac disease on a gluten-containing diet and is therefore used to rule out celiac disease [30]. A total serum IgA test is used to detect whether or not a patient has an IgA deficiency, which is associated with celiac disease and can result in a false negative tTg-IgA finding [30]. Esophagogastroduodenoscopies (EGDs) and colonoscopies are sometimes administered for pediatric patients with GI complaints for the diagnosis of a range of organic GI conditions, like IBD and celiac disease [31]. They allow for the visualization of the digestive tract and can be used to obtain biopsies [31]. Tests were offered at the discretion of each patient’s gastroenterologist to rule out IBD and were individualized based on the patient’s presenting symptoms. In general, patients with FC concentrations > 250 µg/g and epigastric abdominal pain in the absence of weight loss and anemia were also routinely administered an EGD.

### Data analytic approach

Data were analyzed using SPSS version 27 [32]. The data are available from the corresponding author on reasonable request. Item level data were available for all participants but one that was missing SCARED data; thus, no statistical adjustments for missing data were made. Descriptives were used to examine demographic and clinical characteristics and to categorize youth by presence of clinical elevations in anxiety, disability, and pain (ranging from 0 to 3) [7]. Normality of data was assessed. As expected, FC concentration was negatively skewed so non-parametric analyses were used. FC concentration was dichotomized into normal (< 50 µg/g) and elevated (≥ 50 µg/g). Fisher’s exact tests were performed to examine the relation between those with three risk factors versus fewer risk factors in FC elevation. Exploratory Fisher’s exact analyses were conducted to examine differences in FC elevation between other risk groups. Exploratory analyses were also conducted to evaluate the relationships between FC concentration and socioeconomic index (SEI) and type of abdominal pain. Given that additional clinical tests were not given to the entire sample, we were unable to evaluate their associations with FC elevation.

## Results

### Demographic and clinical characteristics

Demographic and clinical data for the sample are presented in Table 1. The majority of the sample was Caucasian (*n* = 50, 89.3%) and female (*n* = 37, 66.1%) and the mean age was 12.23 ± 2.71 years. There were no significant differences between the risk groups with regards to age [*H*(3) = 4.346, *p* = 0.226], gender [*X*^2^(3) = 2.738, *p* = 0.434], SEI [*H*(3) = 3.751, *p* = 0.290], or abdominal pain location (*p* = 0.902, Fisher’s exact test, Cramer’s V = 0.255). The mean FC concentration for the total sample was 51.20 ± 71.44 µg/g, which is considered in the borderline range. Overall, 75% of participants had normal FC concentrations (*n* = 42) and 25% had elevated FC concentrations (*n* = 14). FC concentration did not differ by age (*r*_s_ = 0.068, *p* = 0.620), gender [*H*(1) = 1.187, *p* = 0.276], SEI (*r*_s_ = − 0.265, *p* = 0.109), or type of abdominal pain [*H*(4) = 7.668, *p* = 0.104].

**Table 1:** Sample Characteristics

Variable (total possible range)	% (n)	Mean^a^ (SD)	Clinical reference range
Demographics			
Age in years (9–14)	–	12.23 (2.71)	–
Sex			
Male	33.9 (19)	–	–
Female	66.1 (37)	–	–
Race/ethnicity			
Caucasian	89.3 (50)	–	–
African American	3.6 (2)	–	–
Asian American	3.6 (2)	–	–
American Indian/Alaskan Native	1.8 (1)	–	–
Hispanic/Latinx	1.8 (1)	–	–
Abdominal pain location			
Generalized	51.3 (28)	–	–
Periumbilical	33.3 (19)	–	–
Epigastric	10.3 (6)	–	–
Right lower quadrant	2.6 (1)	–	–
Left lower quadrant	2.6 (1)	–	–
FC^a^	100.0 (56)	20.0 (71.44)	≥ 50 µg/g
Psychosocial risk factors (0–3)			
SCARED (0–82)	98.2 (55)	32.25 (18.73)	≥ 25
FDI (0–60)	100.0 (56)	16.66 (9.58)	≥ 13
NRS Pain (0–10)	100.0 (56)	4.24 (1.84)	≥ 4
Additional diagnostic work-up			
ESR^a^	62.5 (35)	7.50 (8.12)	> 15.0 mm/h
CRP^a^	62.5 (35)	0.29 (0.69)	> 1.0 mg/dL
Albumin	57.1 (32)	4.11 (0.43)	< 3.5 or > 5.5 gm/dL
IgA	64.3 (36)	121.29 (56.37)	< 68.0 or > 378.0 mg/dL
tTG-IgA	64.3 (36)	3.89 (1.43)	> 19 CU
EGD	19.6 (11)	–	–
Colonoscopy	21.4 (12)	–	–

Additional diagnostic work-up was offered at the discretion of the GI provider

*SCARED* Screen for Child Anxiety and Related Disorders; *FDI* Functional Disability Inventory; *NRS* Pain Numeric Rating Scale; *FC* fecal calprotectin; *ESR* erythrocyte sedimentation rate, *CRP* C-reactive protein; *IgA* immunoglobulin A; *tTG-IgA* anti-tissue transglutaminase antibodies

^a^Non-normally distributed, therefore median value is presented. Clinical ranges of the diagnostic tests are standards used in clinical care in the gastroenterology division at Cincinnati Children’s Hospital Medical Center

### Risk status

**Fig. 1:**
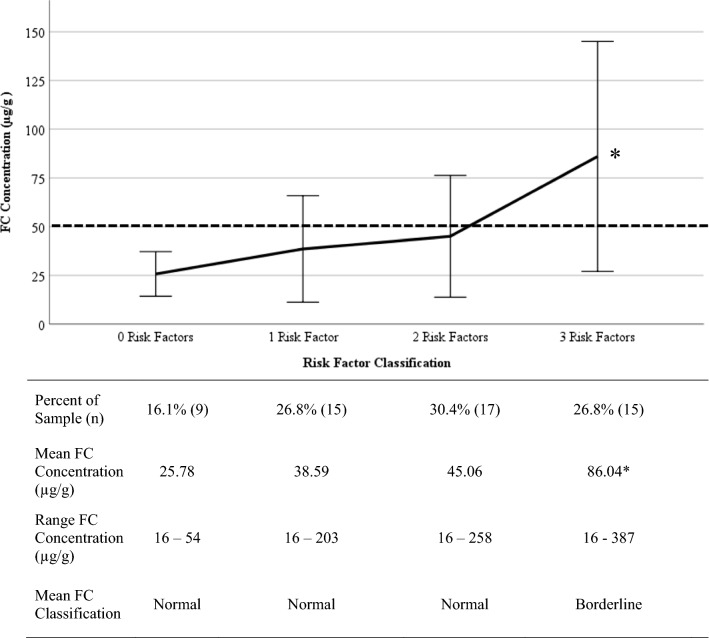
FC concentrations by risk factor classification. *Borderline FC concentration 50.1–120.00 μg/g. Children with three risk factors were more likely to have elevated FC concentrations compared to children with one (*p* = 0.025, one-tailed Fisher’s exact test, Cramer’s V = 0.424) and two risk factors (*p* = 0.040, one-tailed Fisher’s exact test, Cramer’s V = 0.375). The differences in FC concentrations between those with zero, one, and two risk factors were not significant (all *p*’s > 0.05)

Participants were classified into four groups by number of risk factors based on clinical elevations: zero (16.1%), one (26.8%), two (30.4%), and three (26.8%; Fig. 1). Children with three risk factors were significantly more likely to have elevated FC concentrations than those with zero risk factors (*p* = 0.048, one-tailed Fisher’s exact test, Cramer’s V = 0.422). Similarly, children with three risk factors were more likely to have elevated FC concentrations compared to children with one (*p* = 0.025, one-tailed Fisher’s exact test, Cramer’s V = 0.424) and two risk factors (*p* = 0.040, one-tailed Fisher’s exact test, Cramer’s V = 0.375). There were no significant differences in FC elevations between those with zero, one, and two risk factors (all *p*’s > 0.05).

Those with three risk factors (i.e., the most clinically complex manifestation of FAPD) had greater average levels of FC than those with two or fewer risk factors. There were no significant differences in FC elevations between those with zero, one, or two risk factors (all *p*’s > 0.05).

### Exploratory analysis

Given the scope of the study, all participants who underwent an EGD as part of their clinical care had unremarkable endoscopy findings. Existing clinical test and EGD data for participants were gathered via chart review. Overall, 11 participants had EGD with routine biopsies. Twelve had colonoscopies with ileal intubation and routine biopsies, which were all within normal limits. Exploratory analyses revealed no significant differences in the other clinical test values by risk status groups (*p*’s > 0.05).

## Discussion

The present study evaluates a biomarker of gastrointestinal inflammation in pediatric FAPD and demonstrates significantly associated elevated FC amongst those with a clinically complex FAPD profile (elevations in anxiety, disability, and pain) compared to those with two or fewer elevations. Few studies have examined FC in pediatric FAPD [13]. While a recent study detected an association between low-grade inflammation and increased pain interference in pediatric FAPD, [13] no study has examined how FC relates to high risk FAPD. This is an important area of investigation because it is already known that the high risk FAPD group (those categorized by elevations in anxiety, disability, and pain) is more likely to exhibit persistent disability [7].

Use of clinical data available through the EMR yielded meaningful information in this exploratory study. Future research efforts should seek to replicate our findings in larger, prospective trials. Although FC is the most commonly researched indicator of inflammatory response, levels of other GI inflammation biomarkers (e.g., fecal lactoferrin, S100A12, polymorphonuclear elastase, M2-PK [33]) should be evaluated. Additionally, it will be important to examine if GI inflammation differs by FAPD subtype and abdominal pain location in larger scale studies.

The study findings are potentially important for forming a stronger biopsychosocial conceptualization of pediatric FAPD. By defining a phenotype of complex pediatric FAPD characterized by clinically elevated levels of anxiety, disability, and pain, [7] in addition to increased FC levels, we may be better able to better identify youth with persistent FAPD and ultimately develop more targeted and effective treatments.

## Limitations

- The sample was relatively small with an unequal gender distribution in this exploratory investigation.
- Due to the use of real world clinical data, some children received more investigations than others prior to receiving a diagnosis of FAPD from their gastroenterologist.
- Collecting clinical data limited our ability to monitor/control for other factors (e.g., administration of other clinical tests, patient consumption of corticosteroids or mesalamine anti-inflammatory medications before the time of assay) [33] that might affect FC levels.
- Caucasian children may be more likely to receive specialized care for FAPD, contributing to overrepresentation in research conducted in these settings [15, 34, 35] and in our study specifically.

## Data Availability

The datasets used and/or analyzed during the current study are available from the corresponding author on reasonable request.

## Abbreviations

FAPD: Functional abdominal pain disorders
GI: Gastrointestinal
FC: Fecal calprotectin
EMR: Electronic medical record
IBD: Inflammatory bowel disease
SCARED: Screen for Child Anxiety Related Disorders
FDI: Functional Disability Inventory
NRS: Numeric Rating Scale
ESR: Erythrocyte sedimentation rates
RP: C-reactive protein
IgA: Immunoglobulin A
tTG-IgA: Anti-tissue transglutaminase antibodies
EGD: Esophagogastroduodenoscopy
SEI: Socioeconomic index
